# Impact of Rehabilitation on Gait Kinematic following Grade II Anterior Cruciate Ligament Injury among Wrestlers

**DOI:** 10.1155/2022/4895234

**Published:** 2022-05-27

**Authors:** Mohd Arshad Bari, Hussein Ali Hassan Alghazal, Shibili Nuhmani, Ahmad H. Alghadir, Mohd Bilal Tafseer, Amir Iqbal

**Affiliations:** ^1^Department of Physical Education, Aligarh Muslim University, UP, 202002, Aligarh, India; ^2^Department of Physical Therapy, College of Applied Medical Sciences, Imam Abdulrahman Bin Faisal University, Saudi Arabia; ^3^Department of Rehabilitation Sciences, College of Applied Medical Sciences, King Saud University, P.O. Box 10219, Riyadh 11433, Saudi Arabia; ^4^Department of Ilmul Avida, Aligarh Muslim University, UP, 202002, Aligarh, India

## Abstract

**Background:**

Anterior cruciate ligament (ACL) injuries are among the most common injuries in wrestling. Even though there are several studies available in the literature about the changes in gait kinematics following ACL injury and ACL reconstruction surgery, none of these studies investigated the changes in gait kinematics following nonoperative rehabilitation protocol. So, this study is aimed at investigating the changes in gait kinematic following a supervised ACL rehabilitation protocol among wrestlers following a grade II ACL injury.

**Methods:**

Fifteen male professional wrestlers with recent grade II ACL injury with mean age: 19.93 ± 2.01 years, weight: 72.33 ± 7.46 kg, and height: 173 ± 4.95 cm volunteered for this single-arm pretest-posttest study. Kinematic parameters during walking pre- and postrehabilitation were examined by two-dimensional (2D) video graphic analysis. Paired sample *t*-test and Cohen's *d* were used to determine significant differences and effect size of segmental angle, cadence, step length, stride length, etc.

**Results:**

Injured wrestlers after the rehabilitation program walked significantly faster and had a 10.13% higher cadence, a 10.89% faster gait velocity, a 05% greater step length, and a 4.69% longer stride length, compared with a prerehabilitation program of injured wrestlers. Furthermore, joint angles at the hip, knee, and ankle were significantly different between pre- and postrehabilitation.

**Conclusion:**

Research findings suggest that rehabilitation programs significantly impact the gait pattern of injured wrestlers. A 19-week supervised rehabilitation protocol can increase gait velocity and related parameters in ACL injured wrestlers.

## 1. Introduction

Wrestling is considered one of the most challenging sports globally, consisting of quick attack and defense moves that require high muscle power and force with an injury rate of 70 per thousand athletic exposures [1]. Compared to other sports, wrestling gets relatively less attention in terms of injuries. Epidemiological studies reported that wrestling does not cause as many injuries as soccer and taekwondo. At the same time, any part of the wrestler's body is prone to injury, with 65% of the injuries requiring surgery reported in the knee, according to a previous study [2]. According to Ransone et al. [3], knee injury remains the most common injury in wrestling. Knee injuries frequently occur during the takedown or bottom positions, causing damage to collateral ligaments, meniscus, patella, and ACL. Among the reported knee injuries in wrestlers, up to 9.1% are ACL-related injuries [3].

It has been reported in previous studies that the gait pattern may affect following ACL injuries [4–6]. The ACL injury may result in loss of knee joint stability and abnormal movement patterns, leading to the early development of degenerative changes such as osteoarthritis of the knee joint [7]. Evidence suggests nonoperative management (rehabilitation) as a viable alternative to operative management (ACL reconstruction surgery) and reported good functional outcomes [8, 9]. Conversely, biomechanical outcomes following ACL rehabilitation are not well understood. Compared to the uninvolved limb, the patient will walk with lower peak knee joint angle and moments that are obvious in the sagittal plane [10, 11]. It has been reported that patients with acute ACL injury (less than one month) produce a substantially distinct gait pattern from chronic ACL deficient (>2years postinjury) subjects [12, 13]. ACL injury patients may develop a “quadriceps avoidance” strategy to decrease anterior shear during gait.

A meta-analysis conducted by Culvenor et al. [10] reported no difference in peak knee flexion angle and movements after 1-3 years of ACL injuries. Button et al. [14] reported no difference in sagittal plane knee angle and movements in patients who undergone ACL rehabilitation compared to healthy controls. According to Khandha et al. [4], the patient who received rehabilitation protocol following ACL injury walked with 28% greater medial compartment contact forces and 28% greater peak knee adduction moment in the involved knee compared with patients who have undergone ACL reconstruction surgery. Even though there are several studies available in the literature about the changes in gait kinematics following ACL injury and ACL reconstruction surgery [11, 15], none of these studies investigated the changes in gait kinematics following nonoperative ACL rehabilitation protocol. So, this study is aimed at investigating the changes in gait kinematic following a supervised ACL rehabilitation protocol among wrestlers following a grade II ACL injury.

## 2. Materials and Methods

### 2.1. Sample

Fifteen male professional wrestlers from the republic of Iraq with recent grade II ACL injury age: 19.93 ± 2.01 years, weight: 72.33 ± 7.46 kg, and height: 173 ± 4.95 cm volunteered for this single-arm pretest-posttest longitudinal study. The sample size was calculated as 13 using a sample size calculator (https://www.ai-therapy.com/psychology-statistics/sample-size-calculator) based on a previous study [16] that investigated the gait parameters in ACL deficient knees with a 0.76 effect size 0.05 significant level and 0.8 statistical power. A 15% of the sample (2 participants) was added anticipating dropouts which made the sample size 15. However, there were dropouts in the study. The participants were recruited from the local wrestling federations registered under the Iraqi Wrestling Federation. The grade II ACL injury was confirmed by clinical assessment (Lachman test and anterior drawer test) and MRI scan by a senior orthopedic surgeon. Patients who have undergone ACL reconstruction, previous knee surgery, associated injuries to other ligaments or meniscus, and biomechanical abnormalities of the lower limb were excluded from the study. The participants were informed about the design, procedure, advantages, and disadvantages of the participation, and a written informed consent was taken from them. The study was approved by the ethical research committee of Aligarh Muslim University (IRB no: 00/12/ss) and conducted as per the declaration of Helsinki.

### 2.2. The Experimental Approach to the Problem

Prior to the baseline measurement, all the participants have undergone a three-week initial rehabilitation following the ACL injury (acute phase). The initial rehabilitation focused on eliminating the residual symptoms such as pain, effusion, and reducing the impairment. All the participants have undergone initial rehabilitation under the same physical therapist and followed the same rehabilitation protocol. Details of the initial rehabilitation are available in supplementary Table 1. The baseline measurement was taken in the fourth week following the approval of the treating orthopedic surgeon and physical therapist. The criteria to perform the baseline measurement were full ROM of the knee joint, no joint effusion at the knee joint, and no joint line or patella-femoral pain.

### 2.3. Rehabilitation Protocol

The participants underwent a 19-week progressive rehabilitation protocol under the supervision of a senior physical therapist with experience in ACL rehabilitation. All the participants have undergone a similar rehabilitation protocol consistent with the literature consensus, and the details of the protocol are available in Table 1. The rehabilitation was primarily aimed to address the impairment, achieve functional stability, decrease the risk of reinjury, and return to sports activity [16, 17]. Neuromuscular training and perturbations are implemented in the second phase of rehabilitation. The final phase of rehabilitation focused on optimizing the neuromuscular strength and returning the athlete to the preinjury sports level by sports-specific training and improving the psychological readiness for sports participation.

### 2.4. Video Graphic Analysis

All of the A.C.L. grade-II injured wrestlers' gait patterns were captured by using two synchronized Nikon D-7000 video cameras in a field setting at the testing venue for a two-dimensional gait analysis (GA) examination. The first camera was positioned approximately 8.5 meters perpendicular to the sagittal plane and parallel to the mediolateral axis on their walking side (camera optical axes perpendicular to the sig plane), giving a 90° angle between their respective optic axes. The second camera was positioned (08) eight meters behind the stationary position, in an initial position with the camera's optical axis perpendicular to the frontal plane for measuring the upper and lower body segment motion of subjects during various phases of gait. Cameras were also raised (1.1 m) and tilted to get the biggest picture possible while keeping all of the things that were important in the picture in motion.

According to Davis et al.'s procedure [18], twenty-three passive markers were put on the subject's body. Each participant was asked to walk at their own pace on a 05-meter path. Each subject's starting position was selected and located on the pathway to reach the first platform on the right foot and the second platform on the left foot. The video cameras were set to sports mode with a sampling rate of sixty (60) fields per second. The camera's shutter speed was set at high speed (1/2000 fast shutter speed enables rapid-moving subjects). A higher shutter speed will freeze the motion of a fast-moving image, whereas a slow shutter speed will blur the image to create the illusion of motion. Then, for each ACL, injured wrestlers performed three trials on a given specified area. For the following analysis, only the best trials were considered for further examination. The identified trails were played with the help of the software Silicon Coach Pro 8 (SCP) to make separate clips of each player for separate gait phases. This software provides identification of angles, cadence (steps min-1), gait velocity (m s-1), step length (m), and stride length (m) and was considered as spatiotemporal parameters.

### 2.5. Outcome Measures

The following outcome measures were taken for analysis: (1) pre and postrehabilitation measurement of joint angles of the hip, knee, and ankle joint at each stage of swing (initial contact, loading response, mid stance, terminal stance, and toe-off) and stance phase (acceleration, midswing, and terminal swing) of the gait cycle. (2) Pre and postrehabilitation measurement of spatiotemporal parameters of the gait which includes cadence, gait velocity step length, and stride length.

### 2.6. Data Analysis

SPSS (v24.0; IBM Corporation, Armonk, NY, USA) was used for analyzing the data. The normal distribution of the data was confirmed by the Shapiro-Wilk test (*p* > 0.05), and the equality of the variances was satisfied by the Levene test (*p* 0.05). Paired sample *t*-test was used to determine significant differences in segmental angle, cadence, step length, stride length, etc., and used Cohen's *d* to calculate the effect size. The effect size was considered small, medium, and large if Cohen's *d* value was equal to 0.20, 0.50, and 0.80, respectively. A statistically significant difference was defined as a *p* value less than 0.05.

## 3. Results

The primary goal of this study was to determine the clinical impact of a rehabilitation program on specified kinematics of GAIT patterns in professional wrestlers with ACL (level-II) injury. A homogeneously distributed sample was identified using the Shapiro-Wilk test for normal distribution. The Paired Samples *t*-test was carried out to evaluate the significant mean variations in kinematics parameters of gait comparing injured wrestlers' before and postrehabilitation programs. Most of the kinematics parameters were significantly different between pre- and postrehabilitation program. (Tables 2–4).

The Paired Samples *t*-test was used to investigate the mean differences in joint angles (ankle joint, knee joint, and hip joint) at the stance phase of gait between the pre- and postrehabilitation programs of injured wrestlers. The results of Table 2 reveal that kinematic variables, hip joint angles at initial contact (*t* = 2.85, *p* ≤ 0.05), loading response (*t* = 4.1, *p* ≤ 0.05), midstance (*t* = 3.23, *p* ≤ 0.05), terminal stance (*t* = 4.17, *p* ≤ 0.05), and preswing phase (*t* = −3.80, *p* ≤ 0.05) showed significant mean differences between pre- and postrehabilitation programs. The values of hip joint angles Cohen's *d* were 0.74 (*d* > 0.50 − <0.80), which indicate medium effect size at initial contact phase, and 1.05 (*d* > 0.80) at loading response, 0.88 (*d* > 0.80) at midstance, 1.80 (*d* > 0.80) at the terminal stance, and 0.98 (*d* > 0.80) at preswing phase indicate high effect size, respectively. Knee joint angles at initial contact (*t* = 4.29, *p* ≤ 0.05), loading response (*t* = 5.7, *p* ≤ 0.05), midstance (*t* = 5.67, *p* ≤ 0.05), terminal stance (*t* = 11.47, *p* ≤ 0.05), and preswing phase (*t* = −6.57, *p* ≤ 0.05) showed significant mean difference between pre- and postrehabilitation programs. The values of knee joint angles Cohen's *d* were 1.11 (*d* > 0.80) at initial contact, 1.50 (*d* > 0.80) loading response, 1.46 (*d* > 0.80) midstance, 2.4 (*d* > 0.80) terminal stance, and 1.70 (*d* > 0.80) preswing phase, which indicate high effect size, respectively. Ankle joint angles at loading response (*t* = 4.1, *p* ≤ 0.05) and midstance phase (*t* = 5.67, *p* ≤ 0.05) showed significant mean differences between pre- and postrehabilitation programs. The values of knee joint angles Cohen's *d* were 1.05 (*d* > 0.80) loading response and 3.30 (*d* > 0.80) midstance, which indicates high effect size, respectively. Joint kinematic parameter revealed significant differences between pre- and postrehabilitation program in hip, knee, and ankle angles at stance phase with exception ankle angles at initial contact, terminal stance, and preswing phase. In particular, ACL injured wrestlers showed statistically significant change ROM at hip and knee joint compared to prerehabilitation program of injured wrestlers.

The Paired Samples *t*-test was used to investigate the mean differences in joint angles (ankle joint, knee joint, and hip joint) at the swing phase of gait between the pre- and postrehabilitation programs of injured wrestlers. The results of Table 3 reveal that kinematic variables, hip joint angles at acceleration (*t* = 3.72, *p* ≤ 0.05), midswing (*t* = 5.24, *p* ≤ 0.05), and terminal swing phase (*t* = 6.38, *p* ≤ 0.05) showed significant mean differences between pre- and postrehabilitation programs. The values of hip joint angles Cohen's *d* were 0.97 (*d* > 0.80) at acceleration, 1.35 (*d* > 0.80) at midswing, and 1.62 (*d* > 0.80) at terminal swing phase, which indicate high effect size, respectively.

Knee joint angles at acceleration (*t* = 5.94, *p* ≤ 0.05), midswing (*t* = 6.95, *p* ≤ 0.05), and terminal swing phase (*t* = 9.46, *p* ≤ 0.05) showed significant mean differences between pre- and postrehabilitation programs. The values of knee joint angles Cohen's *d* were 1.52 (*d* > 0.80) at acceleration, 1.59 (*d* > 0.80) at midswing, and 2.45 (*d* > 0.80) at terminal swing phase, which indicate high effect size, respectively. Ankle joint angles at the acceleration phase (*t* = 3.18, *p* ≤ 0.05) showed significant mean differences between pre- and postrehabilitation programs. The values of knee joint angles Cohen's *d* were 0.82 (*d* > 0.80) at acceleration which indicates a high effect.

Paired Samples *t*-test was used to investigate the mean difference of spatiotemporal parameter between pre- and postrehabilitation program of injured wrestlers. Results of Table 4 reveal that kinematic variables, cadence (*t* = −4.80, *p* ≤ 0.05), gait velocity (*t* = −5.41, *p* ≤ 0.05), step length (*t* = −4.15, *p* ≤ 0.05), and gait stride length (*t* = −4.01, *p* ≤ 0.05) were showed statistically significant means differences exist between pretest and posttest of rehabilitation program of injured wrestlers. The spatiotemporal parameter Cohen's *d* was 1.24, 1.55, 1.06, and 1.03 (*d* > 0.80), which indicated a large effect size.

ACL injured wrestlers walk postrehabilitation program with a 10.89% increase gait velocity, with a 10.13% increase cadence, with a 5% longer step length and 4.69% greater stride length than prerehabilitation program. Furthermore, postrehabilitation program injured wrestlers showed significantly higher values in the prerehabilitation program, but all gait kinematics values did not reach the values of healthy subjects [19, 20].

## 4. Discussion

The rehabilitation of the ACL is a challenging task. Many therapeutic and sports scientific techniques are presently accessible. However, their usefulness is being scrutinized. Experimental studies were designed to find out the clinical implications of rehabilitation on the gait pattern of ACL grade-II injured wrestlers. Our study showed that a designed rehabilitation program is more effective in improving the gait pattern of injured wrestlers. The wrestlers' gait pattern shows significant changes in joint angles at different phases. Our results for joint angles at various gait speeds were not similar to previous researches [21, 22], which indicates that the significance in our dataset and highlights that our participants are representative of a nonhealthy population [22]. This combined dataset of joint angles could be useful for future work to compare with clinical cohorts and investigate the impact rehabilitation programs on body kinematics during walking have on clinical outcomes.

Hip joint flexion/hyperextension changes reported after the rehabilitation program at initial contact 18.87° to 21.33° (13.08%↑), loading response 19° to 14.8° (22.10%↓), midstance 2.33° to 1.20° (48.49%↓), terminal stance 14.06° to 18.20° (29.37%↑), preswing 12.20° to 15.53° (27.79%↑), initial swing/acceleration phase 16.26° to 19.93° (22.50%↑), midswing 23.86° to 23.86° (38.26%↑), and terminal swing 24.60° to 32.93° (33.86%↑) in line with the studies of Winter [23] showed that 20° hip flexion at initial contact, loading response 15°, midstance 0°, terminal stance 10-20°, preswing 10-20°, initial swing 20°, midswing 30°, and terminal swing 30° in the normal population. It can be seen that hip joint angles achieved maximum flexion due to rehabilitation program 18.87° to 21.33° (13.08%↑) around at initial contact with medium effect size at 0° of the gait cycle and reach most extended with high effect size in terminal stance position 14.06° to 18.20° (29.37%↑) and preswing phase 16.26° to 19.93° (22.50%↑) at about 50% of the gait cycle. Standard normal walking, hip ROM approximately 20° flexion to 20° extensions [23]. In the prerehabilitation program, the ACL injured wrestlers recorded hip joint flexion of 18.87° and extensions of 14.06° deviated from the standard norm [16]. The results of the study showed that the rehabilitation program played a significant role in normalizing hip joint ROM as per the standard normal population [23].

Knee joint flexion changes reported after the rehabilitation program of gait in ACL injured wrestlers at initial contact 3.20 to 1.33° (58.43%↓), loading response 18.33° to 15.06° (17.78%↓), midstance 8.93° to 5.20° (41.76%↓), terminal stance 6.86° to 1.80° (73.76%↓), preswing 24.13° to 32.73° (35.64%↑), initial swing 45° to 55.06° (21.46%↑), midswing 23° to 32.26° (36.34%↑), and terminal swing 5.86° to 0.33° (94.30%↓), in line with the study of reported that flexion at initial contact 0°, loading response 15°, midstance 5°, terminal stance 0°, preswing 30°, initial swing 60°, midswing 30°, and terminal swing 0° in normal population [16]. The knee joint standard normal ROM reported by previous researches from 0° (straight) to 60° flexion [23]. Knee joints reached approximately straight position with high effect size 3.20° to 1.33° at initial contact phase and nearly straight again (6.86° to 1.80°) just before hell off at 40% of gait cycle [24–26].

During the swing phase, knee joint angles reached their maximum flexion with a high effect size of 45° to 55.06° of 70% of the gait cycle. Small knee flexion phases occur at 10 to 20% of the phase of the gait cycle. The study results show significant differences between the pre- and postrehabilitation program on knee joint ROM of the injured wrestlers, and the rehabilitation program played a significant role in normalizing knee joint ROM as per the standard norm reported [23]. The abovementioned results show that rehabilitation programs play a significant role in normalizing knee joint kinematics and gait patterns in ACL injured wrestlers [24–27].

Ankle joint plantar/dorsal flexion significant changes reported after the rehabilitation program at loading response 5.06° to 5.80° (25.09%), midstance 6.06° to 4.8° (20.79%), and initial swing/acceleration phase 9.06° to 11.66° (21.46%), in line with the study [22] showed that flexion at initial contact 0°, loading response 5°, midswing 0°, and terminal swing. Ankle joint angles reach maximum dorsal flexion of 6.06° to 4.8° at midstance phase at about 30% of the gait cycle and reach maximum plantar flexion of 17.73° to 18.93° at preswing phase (60%) with no effect on size. The ankle joint normal range of motion (ROM) is 7° dorsiflexion to 25° plantar flexion as reported [23]. The study results showed that the rehabilitation program played a significant role in the normalization of the ROM of the hip, knee, and ankle joints.

The cadence of gait was showed significant means differences with large effect size. In the current study, cadence significantly increased 10.13% from 95.33 s/m to 105 s/m. Results of the present study, in line with previous studies, have reported normal walking gait cadence 110 s/m by Boston and Sharpe [28], 111 s/m by Davis et al. [19], 117 s/m by Finley and Cody [29], and 112 s/m by Öberg et al. [30]. The abovementioned results showed that the rehabilitation program significantly affected gait cadence toward its normalization as per the standard normal population reported in the studies. Gait velocity was showed statistically significant means differences with large effect size. In the current study, gait velocity significantly increases 10.89% due to rehabilitation program from 1.01 m/s to 1.13 m/s in line with previous studies that have provided normal walking gait velocities 1.37 m/s [28], 1.22 m/s [29], and 1.34 m/s [30]. Gait step length and stride length were showed statistically significant means differences exist large effect size in gait pattern. In the current study, step length increased 5% from 61.97 cm to 65.07 cm, and stride length increased 4.69% from 128.51 cm to 134.54 cm significantly due to rehabilitation program in line with previous studies that have provided normal walking gait length of one stride 148 cm [28], 123 cm [29], and 141 cm [30, 31].

We acknowledge that our study has some limitations. The study was conducted on male professional wrestlers. So, the result of the study cannot be generalized to other populations. The strength and flexibility of the lower limb muscles and preinjury gait characteristics may influence the gait kinematic following ACL rehabilitation. Due to the nature of the study, the researchers were not able to assess these factors in the current study. Due to ethical reasons, the researchers could not incorporate a control group in the current study. The baseline measurements were taken in the fourth week of rehabilitation after approval from the treating physician and physical therapist. Even though a similar rehabilitation protocol was given to all the participants in the first three weeks of rehabilitation, this might have influenced the baseline measurements. Psychological support and motivation are given to the patient during rehabilitation, and the individual motivation of the patients are important factors that influence the success of the rehabilitation. It is impossible to decide to what extent these factors influenced the rehabilitation outcome in the current study.

## 5. Conclusion

This study has provided that a 19-week supervised rehabilitation program significantly affected gait velocity, stride length, and step length. We believe this study may have potential implications for clinical practice to influence assessment and treatment methods. These findings show that exercise can increase gait velocity and related parameters in ACL injured persons. Future studies with larger sample sizes and longer follow-ups are required to determine the long-term impact of rehabilitation programs on gait kinematics.

## Figures and Tables

**Table 1:** The rehabilitation program (19 weeks) used in the study.

Subacute stage	
Goals	Maintain ROM and flexibility Restore muscle strength and proprioception Improve neuromuscular control
Activity/exercises	Quadriceps sets (10 × 15 sec hold) Leg press (3 sets × 10 rep) Self ROM stretching (3 × 20 − 30 sec. hold) Front and side lunges (3 sets × 10 rep) Step up and squat progression Planks (3 sets × 10 rep) Progressive resistance training Bicycle exercise for ROM (10 minutes) Eccentric quadriceps training (3 sets × 10 rep) Lateral lunges (3 sets × 10 rep) Progressive neuromuscular and proprioceptive drills
Advanced strengthening and neuromuscular control stage	
Goals	Full ROM and flexibility Maximal strength and neuromuscular control Improve balance and proprioception Restore limb function
Exercises	Progressive resistance training Leg press (3 sets × 20 rep) Lateral lunges and step-ups (3 sets × 20 rep) Step down (3 sets × 20 rep) Biodex stability system (stance—bilateral progress to unilateral, setup—dynamic balance, level 8—progress to level 4, duration—5 bouts of 30 sec. progress to 10 bouts) Pool exercises Advanced neuromuscular drills Perturbation training Dynamic stabilisation
Advanced activity stage	
Goals	Normalise strength Improve proprioception, balance, and neuromuscular control Sports specific training
Exercises	Advanced neuromuscular drills Plyometric training (start with a low rep, sets. 3 × 20 reps progress toward 30 reps. progress based on clinical judgment)
Return to sports stage	
Goal	Unrestricted sports activity
Exercises	Sports specific training Running and agility exercises Advanced plyometrics (progress based on clinical judgments) Strength and neuromuscular training

**Table 2:** Comparison of joints angles mean between pre- and postrehabilitation program at stance phase.

Joint angles	Stance phase		Paired differences			*t*	*p* value	Cohen's *d*
		Mean ± SD.	Mean %	SD	SEM			
HA (pre)	Initial contact	18.87 ± 2.85	13.08%**↑**	3.36	0.87	2.85	0.04∗	0.74
H.A. (post)		21.33 ± 1.87						
KA (pre)		3.20 ± 1.65	58.43%↓	1.68	0.43	4.29	0.02∗	1.11ˆ
KA (post)		1.33 ± 0.49						
A.A. (pre)		1.33 ± 0.48	05.26%↓	0.59	0.15	0.44	0.67	0.10
A.A. (post)		1.27 ± 0.46						
HA (pre)	Loading response/foot flat	19.00 ± 3.02	22.10%↓	3.96	1.02	4.1	0.02∗	1.05ˆ
H.A. (post)		14.80 ± 2.21						
KA (pre)		18.33 ± 2.5	17.78%↓	2.18	2.05	5.7	0.01∗	1.50ˆ
KA (post)		15.06 ± 1.33						
A.A. (pre)		5.06 ± 0.88	25.09%**↑**	1.27	-1.44	2.21	0.04∗	0.57
A.A. (post)		5.80 ± 1.37						
HA (pre)	Mid-stance	2.33 ± 1.23	48.49%↓	1.35	0.35	3.23	0.03∗	0.88ˆ
H.A. (post)		1.20 ± 0.41						
KA (pre)		8.93 ± 2.18	41.76%↓	2.54	0.65	5.67	0.01∗	1.46ˆ
KA (post)		5.20 ± 0.86						
A.A. (pre)		6.06 ± 1.27	20.79%↓	1.48	0.38	3.30	0.03∗	0.85ˆ
A.A. (post)		4.80 ± 0.56						
HA (pre)	Terminal-stance	14.06 ± 2.49	29.37%**↑**	3.83	0.98	4.17	0.02∗	1.08ˆ
H.A. (post)		18.20 ± 3.21						
KA (pre)		6.86 ± 1.40	73.76%↓	1.70	0.44	11.47	0.01∗	2.94ˆ
KA (post)		1.80 ± 0.86						
A.A. (pre)		1.93 ± 0.88	0.00%	1.25	0.32	0.00	1.00	0.00
A.A. (post)		1.93 ± 0.79						
HA (pre)	Preswing/toe off	12.20 ± 2.56	27.79%**↑**	3.39	0.87	3.80	0.03∗	0.98ˆ
H.A. (post)		15.53 ± 3.35						
KA (pre)		24.13 ± 2.97	35.64%**↑**	5.06	1.30	-6.57	0.01∗	1.70ˆ
KA (post)		32.73 ± 4.14						
A.A. (pre)		17.73 ± 2.43	-6.76%**↑**	3.54	0.91	1.30	0.21	0.34
A.A. (post)		18.93 ± 2.25						

HA: hip angle; KA: knee angle; AA: ankle angle; df = 14; ∗significant value if *p* ≤ 0.05; SD: standard deviation; SEM: standard error mean; ˆeffect size is large if *d* = 0.8.

**Table 3:** Comparison of joints angles mean between pre- and postrehabilitation program at swing phase.

Joint angles	Swing phase		Paired differences			*t*	*p* value	Cohen's *d*
		Mean ± SD	Mean	SD	SEM			
HA (pre)	Acceleration phase	16.26 ± 3.21	22.50%**↑**	3.81	0.98	3.72	0.03∗	0.97
H.A. (post)		19.93 ± 3.08						
KA (pre)		45.00 ± 6.4	21.46%**↑**	6.29	1.62	5.94	0.01∗	1.52
KA (post)		55.06 ± 5.09						
AA (pre)		9.06 ± 2.01	28.69%**↑**	3.15	0.81	3.18	0.03∗	0.82
A.A. (post)		11.66 ± 2.84						
HA (pre)	Midswing (swing phase)	23.86 ± 3.96	38.26%**↑**	6.73	1.73	5.24	0.01∗	1.35
HA (post)		33.00 ± 4.48						
KA (pre)		23.66 ± 3.67	36.34%**↑**	5.40	1.39	6.15	0.01∗	1.59
KA (post)		32.26 ± 4.28						
AA (pre)		0.46 ± 0.51	71.73%**↑**	0.89	0.23	1.4	0.17	0.37
A.A. (post)		0.80 ± 0.67						
H.A. (pre)	Terminal-swing (swing phase)	24.60 ± 3.15	33.86%**↑**	5.05	1.30	6.38	0.01∗	1.65
H.A. (post)		32.93 ± 4.14						
KA (pre)		5.86 ± 2.06	94.3%↓	2.26	0.58	9.46	0.01∗	2.45
KA (post)		0.33 ± 0.72						
A.A. (pre)		1.26 ± 0.45	0.00	0.65	0.16	0.00	1.000	00.00
A.A. (post)		1.26 ± 0.45						

HA: hip angle; KA: knee angle; AA: ankle angle; df = 14; ∗significant value if *p* ≤ 0.05; SD: standard deviation; SEM: standard error mean; ˆeffect size is large if *d* = 0.8.

**Table 4:** Comparison of spatiotemporal parameter of the study group.

Variables	Mean ± SD	Paired differences			*t*	*p* value	Cohen's *d*
		Mean	SD	SEM			
Cadence (pre) s/m	95.33 ± 5.69	10.13%**↑**	7.78	2.01	4.80	0.02∗	1.24ˆ
Cadence (post) s/m	105.00 ± 5.16						
Gait velocity (pre)	1.01 ± 0.12	10.89%**↑**	0.08	0.02	5.41	0.01∗	1.55ˆ
Gait velocity (post)	1.13 ± 0.11						
Step length (pre)	61.97 ± 5.04	5.0%**↑**	2.89	0.74	4.15	0.02∗	1.06ˆ
Step length (post)	65.07 ± 6.87						
Stride length (pre)	128.51 ± 17.62	4.69%**↑**	5.82	1.50	4.01	0.02∗	1.03ˆ
Stride length (post)	134.54 ± 18.67						

∗Significance value if *p* ≤ 0.05; SD: standard deviation; SEM: standard error mean; df: 14; ˆeffect size is large if *d* = 0.8.

## Data Availability

The data set for the result of this study will be available from the corresponding author upon reasonable request.

## Supplementary Materials

Supplementary Materials Supplementary Table 1: the three-week initial rehabilitation protocol used in the study during the acute phase of the injury.
