# Supplementary material for: Impact of Rehabilitation on Gait Kinematic following Grade II Anterior Cruciate Ligament Injury among Wrestlers
Source: Biomed Res Int. 2022 May 27;2022:4895234. doi: 10.1155/2022/4895234 (PMC9167103; doi:10.1155/2022/4895234)
Supplement: Supplementary Materials — Supplementary Table 1: the three-week initial rehabilitation protocol used in the study during the acute phase of the injury. [file 4895234.f1.zip › Supplimetry table 1. Revised.docx]

**Supplementary Table 1 The three-week initial Rehabilitation protocol used in the study during the acute phase of the injury**

| Acute stage |  |  |
| --- | --- | --- |
|  | Goals | Control pain and swelling  Restore full knee extension (passive)  Improve knee flexion ROM  Establish quadriceps control |
|  | Activity / Exercises | Cryotherapy and elevation  Knee brace  Weight-bearing – crunches, as tolerated  Patellar mobilisation (5 min., 4 x/day superior/inferior and medial/lateral glides)  Heel slide (3 sets,10 rep)  SLR (hold 5 sec. 3 sets, 10 rep)  Quadriceps sets (hold 10 sec. 1 set, 10 rep)  Weight shifts  Proprioceptive and balance exercises |
